# Editorial: Enhancing the right to science: the triple planetary crisis and the need for comprehensive approaches

**DOI:** 10.3389/fsoc.2024.1406640

**Published:** 2024-07-08

**Authors:** Peter Bille Larsen, Konstantinos Tararas

**Affiliations:** ^1^Department of Sociology, Environmental Governance and Territorial Development Institute, University of Geneva, Geneva, Switzerland; ^2^Social and Human Sciences Sector, UNESCO, Paris, France

**Keywords:** science, human rights, planetary crisis, Sustainable Development, climate change, academic freedom, science diplomacy, right to science

## Introduction: the right to science and sustainability challenges of our times

Enhancing the right to science is increasingly recognized as a central piece in the multi-facetted puzzle of solving the triple planetary crisis (Orellana, 2021). Its role as a cross-cutting catalyst in relation to other human rights dimensions of major global challenges from pandemics, biodiversity, toxics to climate change, calls for far more comprehensive attention to the bundle of rights linking science, scientists and scientific practice to contemporary sustainability responses (Larsen and Pamintuan, 2022).

The global handling of the COVID-19 pandemic demonstrated with all clarity the added value of leveraging the right to the benefits of science to advance the right to health agenda, reshaping the governance of intellectual property rights and fostering access to life-saving technologies. Countering challenges such as uneven vaccine distribution and access to health services that are tied to deep-running inequalities across and within countries requires the transformation of the entire science ecosystem. This is precisely where the right to science can be instrumental.

More generally, key dimensions of the right, from access to scientific knowledge and methodologies, anticipation of and protection against harm deriving from scientific discovery and technological innovation, to autonomy and freedom of scientific researchers, are critical cross-cutting drivers of the far-reaching positive change needed to face the daunting challenge of the triple planetary crisis.

Yet, there is important ground still to be covered before the right to science can make a difference on the ground. Although calls for academic freedom are common, for example, they are rarely framed in the wider context of the right to science. Still, whereas the right to science long suffered from relative neglect and lack of clarity (Committee on Economic, Social and Cultural Rights, 2020), rapid normative policy developments, stakeholder dialogue and emerging platforms potentially signal a new era both in terms of opportunities and challenges.

As the triple planetary crisis deepens, debates around science-policy platforms, intergovernmental negotiations and shrinking civic spaces both domestically and in global arenas demonstrate the urgency of engaging with the right to science. From standard-setting in the fields of AI to global pandemics, how to reinforce the role of rights-based approaches in the growing field of science diplomacy is critical. On-going dialogues on regulatory siloes, conflicts of interest (Schäffer et al., 2023) and science policy platforms in the field of chemicals (Brack et al., 2022) are a case in point equally relevant when addressing climate change and biodiversity. The triple planetary crisis demonstrates how on-going processes are in dire need to be shaped not only by immediate national imperatives and corporate priorities, but equally so from a public good lens, and clear priorities from a set of converging human rights.

This Research Topic is a unique entry point to better understand these interlinked frontiers of human rights, science and sustainability challenges. Firstly, it answers the call for further analysis emitted by a multi-stakeholder dialogue seeking to draw out emerging trends, institutional challenges and opportunities. The 2022 Geneva dialogue on the right to science, in particular, made the paradox of the right to science in the contemporary world abundantly clear (https://www.unesco.ch/francais-right-to-science-summary-report-and-video-of-the-second-geneva-human-rights-dialogue). Despite its importance, the footprint of the right to science in human rights mechanisms and transversal reporting has been extremely limited (Swiss Commission for UNESCO, 2022), including in the Committee on Economic, Social and Cultural Rights, where the follow-up to its General Comment N. 25 (Porsdam and Porsdam Mann, 2021) remains partial. Participants felt that for the right to science to be systematically prioritized in advocacy, policy making and regulatory practice, it would be necessary to expand reflection and the elaboration of operational guidance. Secondly, the Research Topic brings together a number of leading researchers and practitioners whose opinion pieces shed light on the scope and complexity of the right to science. If the right to science is articulated in singular, its implications are, in both normative and practical senses, multiple and far-reaching.

## Leveling the playing field in and through the right to science

This Research Topic points to the global inequalities and fragile foundations shaping contemporary scientific practice. In large parts of the global South researchers are today struggling to maintain independently-financed sustainability agendas and with adequate laboratories, academic freedoms and well-resourced research programmes. These are not merely matters of national public policy, but concern how access has been reshaped by commercial contracting and publishing practices in the academic sphere. As Beiter underlines in his piece, the tension between copyright and the ability to access, use or reuse scholarly publications, impedes the access to science and its benefits. Uneven access and inequalities are particularly glaring in the global South, leading Beiter to challenge the political economy of commercialized science, and call for open science to ensure that knowledge commons are global in scope. Echoing on-going critique and renegotiations between funders, the research community and commercial publishers, he calls for free publishing instead of heavy fees, open science and fair compensation to publishers. In his view, a new global contract enabling the “free flow of knowledge for all, within the global North, within the global South, and between global North and global South” would be the solution.

Yet, what about the uneven recognition and inequalities between different knowledge systems and practices? Gatt, in her opinion piece, stresses the need to decolonize scholarship and science in a call for plural epistemologies. Starting from the assumption that research may perpetuate coloniality, Gatt challenges us to rethink the role of science in perpetuating “epistemic violence”, underlining the importance of other ways of knowing. This also echoes recent calls from the UN Special Rapporteur in the field of cultural rights for emphasizing participation, a decolonized understanding and giving due consideration to “scientific diversity” (Xanthaki, 2024).

In addition, as Donders underlines in her opinion piece, gender inequalities are persistent and deep-running in the fields of education, sciences and research. Questions of access, participation, the contribution to and enjoyment of the benefits of science have critical gender dimensions sustaining wider inequalities. The right to science can be the basis for a comprehensive response.

Finally, the right to science is gaining growing importance as a stronghold against the hollowing out of independent research, a weakened, sometimes toxic, media landscape and the expansion of populism and polarized politics. Porsdam, in this issue, underlines the risks with disinformation, anti-science skepticism and “science-related populism”, which threaten the very space available for sound research and scientific contributions to societal problem solving. In a social media landscape where fake news may pitch science as elitist, effective standards, freedom of expression and guarantees are urgently needed to protect scientific community and practice.

## Global challenges and specific insights

Faced with inter-related crises of planetary dimensions, global approaches, cooperation and safeguards are critical building blocks.

The relationship of law, ethics and rights when addressing issues of science and technology would require rebalancing, argues Murphy calling for further human rights literacy in professional bodies and ethics committees.

This is also true, in terms of maintaining spaces for international cooperation as explicitly recognized the “benefits to be derived from the encouragement and development of international contacts and co-operation in the scientific field” in Article 15(4) of the Covenant on Economic, Social and Cultural Rights. With investment in research and development concentrated in a handful of countries with a focus on Northern priorities, international cooperation is yet to be fully mobilized in the spirit of the right to science. Achermann and Besson consider the current indeterminacies surrounding the duty of international cooperation as a major obstacle that needs to be dispelled. They propose two complementary actions: articulating clear legal duties and responsibilities; and building international institutions to co-specify and co-allocate duties and responsibilities between their multiple bearers.

What then are the lessons from recent global responses? In the aftermath of the COVID-19 pandemic, ongoing discussions on “pandemic prevention, preparedness, and response” (PPPR) seem to recognize the value of the right to science and foundational concepts and principles. While considered a positive development, Dang and Frick here argue that this is not enough. They underline how “the right to science should stand as a distinct guidepost for human rights-based governance of global health challenges”, stressing its importance in fostering more equitable international cooperation, access to health technologies and information, and participation in science and decision-making.

Weisenberg also argues that the right to science, as the COVID-19 pandemic demonstrated, can become a solid point of reference for accelerated action against the ongoing interlocking crises, including the “triple planetary crisis” of climate change, biodiversity loss, and pollution. This is illustrated by the potential of the right to address multiple, interlocking issues; to promote public access to, and participation in, science; and to strengthen international scientific cooperation and the international science-policy interface.

## Good omens from the intergovernmental arena

A number of developments in the intergovernmental arena since the end of 2023—such as the 75th anniversary of the Universal Declaration on Human Rights—are a legitimate source of optimism that the path does not stop here. On the part of UNESCO, normative work in the domain of science continues at full speed. The 42nd session of the Organization's General Conference decided to embark on a new instrument on the ethics of neurotechnologies, for example (42 C/Resolution 29). If recent additions to its normative arsenal are a point of reference (ie. the Recommendation on Open Science and the Recommendation on the Ethics of Artificial Intelligence both adopted in 2021), then the new text will certainly be anchored in human rights, emphasizing the right to science in particular.

This standard-setting fervor is combined with a serious commitment to the operationalization of earlier instruments. For example, new impetus has been given to the Recommendation on Science and Scientific Researchers (2017). An Executive Board decision (UNESCO's Executive Board, 2023) called for stronger implementation of the Recommendation reinforced by a General Conference resolution (UNESCO's General Conference, 2023), which mandated the establishment of a programme on freedom and safety of scientific researchers.

The new programmatic framework expands UNESCO's work in favor of the free flow of ideas beyond journalists and artists by putting the spotlight on a key, but insufficiently addressed, aspect of the right to science. The creation of open and safe science ecosystems where scientists can operate without fear of retaliation, free from any undue interference and censorship, is a prerequisite for quality and trustworthy scientific outcomes and therefore for a stronger reliance on scientific evidence for policy making.

The Call to Action in relation to this programme welcomed by the Organization's Executive Board in spring 2024 (UNESCO's Executive Board, 2024a) with 60 Member States explicitly supporting could serve in relation to this programme could serve to incentivize governments and all concerned actors toward elevating this agenda at both national and international levels. Moreover, the different action points highlighted in the Call to Action provide a comprehensive blueprint for shaping UNESCO's work in this domain. Finally, the 219th session of the Executive Board could contribute to the uptake of the right to science through another angle; namely through the questionnaire for the second monitoring cycle of the 2017 Recommendation with references to the different dimensions of right to science considerably reinforced (UNESCO's Executive Board, 2024b).

Importantly, UNESCO's continued attention to the articulation of science and human rights is reverberated by other UN forums. The 2023 Social Forum of the UN Human Rights Council focused on the role of science, technology and innovation in the promotion and protection of human rights (https://www.ohchr.org/en/events/forums/2023/2023-social-forum). While not explicit in the title, the attention to the right to science was much stronger in the debates and the conceptualization of its different sessions. Furthermore, it could hardly be considered a coincidence that the UN special rapporteurs in the field of cultural rights and on the right to education have decided to dedicate recent reports to topics falling within the scope of the right to science. Developments in the UN system are reverberated by converging outcomes in other multilateral forums. The EU Commission has since 2022, conducted a multilateral dialogue process on values and principles for international cooperation on research and innovation. The Brussels statement (European Commission, 2024) adopted at a ministerial conference in February 2024 by more than 40 countries from different regions features the promotion and protection of scientific freedom and the safety of scientists and the advancement of the right to science feature among the priorities.

## Concluding remarks; critical priorities, concerns and risks

While departing from different policy areas, the opinion pieces converge in two major conclusions. On the one hand they acknowledge the changing status of the right—moving from oblivion and neglect to a timid, yet, potentially central role in transforming science ecosystems and countering the triple planetary crisis. They concur that a stronger application of the human right to science could become a game changer, leading to a much needed systematic human rights-based approach to science. On the other hand, they suggest that the progress made so far (both the aura surrounding the right in current international debates and the greater normative clarity), while significant, will be insufficient unless accompanied by enhanced implementation efforts.

These efforts should take full advantage of international frameworks with the potential to trigger multi-stakeholder cooperation. The Agenda 2030 and the Sustainable Development Goals remains a powerful instrument despite worrying trends and insufficient human rights entrenchment. The newly proclaimed International Decade on Sciences for Sustainable Development could be another opportunity, particularly if it succeeded in mobilizing the scientific community through a synergistic approach, integrating all sciences, critical voices and domains of knowledge.

Of course, any attempt to advance this agenda would need to address structural problems and opposing trends at the national level coupled with an inauspicious climate for international cooperation. The deep-running inequalities across regions and within countries are such an obstacle. Other hurdles to be overcome are the divide between what is necessary for the wellbeing of society and what is privately profitable, the erosion of trust in science and related polarization, budget cuts, shrinking academic freedoms and precarity, to name but a few.

The message to be drawn from all this is, first and foremost, the need for determination. The importance of the right to science in developing adequate responses to key sustainability challenges of our times—from climate change, pollution and biodiversity loss to global health crises and pandemics—cannot be overstated. Notwithstanding the difficulties surrounding its operationalization, as demonstrated in this Research Topic, states as fundamental duty-bearersshould seize the opportunities created by recent developments to take the extra steps needed to translate international norms into change on the ground.

## Author contributions

PL: Writing – original draft, Writing – review & editing. KT: Writing – original draft, Writing – review & editing.

## References

[B1] Brack W. Culleres D. B. Boxall A. B. Budzinski H. Castiglioni S. Covaci A.. (2022). One planet: one health. A call to support the initiative on a global science–policy body on chemicals and waste. Environm. Sci. Eur. 34:21. 10.1186/s12302-022-00602-6 35281760 PMC8902847

[B2] Committee on Economic Social and Cultural Rights. (2020). General Comment No. 25 on Science and Economic, Social and Cultural Rights (Document E/C.12/GC/25).

[B3] European Commission (2024). Brussels Statement, Multilateral Dialogue on Principles and Values for International Cooperation in Research and Innovation. Brussels: Ministerial Conference.

[B4] Larsen P. B. Pamintuan M. (2022). The Human Right to Science: From Fragmentation to Comprehensive Implementation? No. 163. Research Paper.

[B5] Orellana M. (2021). “Right to science in the context of toxic substances - Report of the Special Rapporteur on the implications for human rights of the environmentally sound management and disposal of and wastes,” in A/HRC/48/61

[B6] Porsdam H. Porsdam Mann S. (2021). The Right to Science Then and Now. Cambridge University Press.

[B7] Schäffer A. Groh K. J. Sigmund G. Azoulay D. Backhaus T. Bertram M. G.. (2023). Conflicts of interest in the assessment of chemicals waste and pollution. Environm. Sci. Technol. 57, 19066–19077. 10.1021/acs.est.3c04213 37943968 PMC10702428

[B8] Swiss Commission for UNESCO (2022). “The right to science: understanding trends in and enhancing the effectiveness of human rights mechanisms and partnership approaches”, in UNESCO and Human Rights: Geneva Dialogues for Enhancing Cooperation and Effectiveness, Swiss National Commission for UNESCO (Geneva: University of Geneva, UNESCO, OHCHR and the REGARD network).

[B9] UNESCO's Executive Board (2023). Strengthening the implementation of the 2017 Recommendation on Science and Scientific Researchers, 216 EX/Decision 45.

[B10] UNESCO's Executive Board (2024a). UNESCO's Programme on the Promotion of Scientific Freedom and the Safety of Scientists and a Related Call to Action, Document 219 EX/30

[B11] UNESCO's Executive Board (2024b). Implementation of standard-setting instruments, Part IV: Implementation of the 2017 Recommendation on Science and Scientific Researchers; Preparations for the Next Consultation, Document 219 EX/17.IV.

[B12] UNESCO's General Conference (2023). Strengthening the implementation of the Recommendation on Science and Scientific Researchers (2017), 42 C/Resolution 26.

[B13] Xanthaki A. (2024). “Right to science - report of the Special Rapporteur in the field of cultural rights”, in *A/HRC/*55/44.

